# A standardized and reproducible protocol for serum-free monolayer culturing of primary paediatric brain tumours to be utilized for therapeutic assays

**DOI:** 10.1038/srep12218

**Published:** 2015-07-17

**Authors:** Emma Sandén, Sofia Eberstål, Edward Visse, Peter Siesjö, Anna Darabi

**Affiliations:** 1 Glioma Immunotherapy Group, Division of Neurosurgery, Department of Clinical Sciences Lund, Lund University, Lund, Sweden; 2 Division of Neurosurgery, Department of Clinical Sciences Lund, Skåne University Hospital, Lund, Sweden

## Abstract

*In vitro* cultured brain tumour cells are indispensable tools for drug screening and therapeutic development. Serum-free culture conditions tentatively preserve the features of the original tumour, but commonly comprise neurosphere propagation, which is a technically challenging procedure. Here, we define a simple, non-expensive and reproducible serum-free cell culture protocol for establishment and propagation of primary paediatric brain tumour cultures as adherent monolayers. The success rates for establishment of primary cultures (including medulloblastomas, atypical rhabdoid tumour, ependymomas and astrocytomas) were 65% (11/17) and 78% (14/18) for sphere cultures and monolayers respectively. Monolayer culturing was particularly feasible for less aggressive tumour subsets, where neurosphere cultures could not be generated. We show by immunofluorescent labelling that monolayers display phenotypic similarities with corresponding sphere cultures and primary tumours, and secrete clinically relevant inflammatory factors, including PGE_2_, VEGF, IL-6, IL-8 and IL-15. Moreover, secretion of PGE_2_ was considerably reduced by treatment with the COX-2 inhibitor Valdecoxib, demonstrating the functional utility of our newly established monolayer for preclinical therapeutic assays. Our findings suggest that this culture method could increase the availability and comparability of clinically representative *in vitro* models of paediatric brain tumours, and encourages further molecular evaluation of serum-free monolayer cultures.

Primary brain tumours, including medulloblastomas, astrocytomas, ependymomas and atypical rhabdoid tumours, are the most common solid tumours in children. Since current therapeutic approaches fail in more than 20% of patients and the survivors acquire long-term cognitive and physical sequels from treatment, new and more specific therapies are urgently needed. Recent transcriptional and epigenetic profiling efforts have defined subgroups with distinct prognosis both within and across paediatric brain tumour entities^1^,^2^,^3^,^4^, underscoring the need for a more personalized analysis and therapy to improve survival in these patients. Relevant model systems that mimic the clinical situation are however scarce; while *in vitro* cultured tumour cells are indispensable preclinical tools for drug screening and therapeutic development, most commercially available brain tumour cell lines have been propagated for decades in cell culture medium containing foetal bovine serum. As a consequence, the original molecular features and biological behaviour of the tumour cells have been seriously altered, and the clinical relevance of the information that can be obtained from them is uncertain^5^,^6^.

Low-passage patient-derived tumour cells have emerged as an attractive alternative to traditional tumour cell lines. Moreover, culturing in serum-free medium, supplemented with epidermal growth factor (EGF) and fibroblast growth factor (FGF), has been shown to better maintain the features of the original tumour, including preservation of tumour antigen expression and tumour-initiating cell (TIC) populations^6^,^7^. The traditional approach to isolating and propagating neural stem cells (NSCs) and brain tumour TICs comprises culturing of cells as neurospheres^8^,^9^,^10^. The neurosphere assay however harbours numerous practical disadvantages; sphere cultures are technically difficult to establish and cannot readily be obtained from all primary tumour types tumours^11^,^12^; cells are difficult to maintain in long-term sphere cultures without differentiation and apoptosis occurring^13^,^14^; spheroid cells are heterogeneous in terms of viability, growth rate and differentiation state and are therefore suboptimal for standardized and reproducible *in vitro* assays. In contrast, monolayer cultures are homogenously exposed to growth factors, nutrients and oxygen, tentatively preventing cell death and differentiation. Attempts have therefore been made to culture human NSCs^15^ and glioblastoma TICs^16^ as monolayer cultures, by utilizing the attachment substrate laminin. A similar approach could hypothetically be feasible for culturing of paediatric brain tumour cells, in order to increase the availability and comparability of clinically representative *in vitro* models.

The immune system harbours the potential to eradicate neoplastic cells by effector cells (NK cells, CD8^+^ T cells, macrophages) and release of soluble factors (interferons, tumour necrosis factors, interleukins, nitric oxides, perforin and granzymes). Overtime, tumour cells however acquire immune-evading properties, including cell-surface expression and secretion of immunosuppressive factors such as prostaglandin E_2_ (PGE_2_), and induction and recruitment of immunosuppressive cell populations (regulatory T cells, myeloid-derived suppressor cells) to the tumour site^17^,^18^. Pro- and anti-immune functions, as well as apoptosis, angiogenesis, cell growth and cell differentiation, are mediated by intratumoural and systemic cytokine signalling^19^. We have previously developed a translational immunotherapy of adult brain tumours, encompassing immunizations with irradiated tumour cells, adjuvant cytokines, local administration of cytostatics and reduction of immune suppression^20^,^21^,^22^,^23^,^24^,^25^,^26^,^27^,^28^. Recent evidence, linking host immunity to the survival of paediatric brain tumour patients^29^,^30^,^31^, suggests that children may also benefit from such a treatment approach. However knowledge about the intricate interactions of cytokine signalling networks in paediatric brain tumour patients is scarce, and required for the design of effective immunotherapies.

Here, we define a simple, standardized, non-expensive and reproducible protocol for establishing and propagating primary paediatric brain tumour cell cultures as monolayers in serum-free medium. We use immunofluorescent protein labelling, secretory cytokine profiling and drug treatment to characterise our newly established *in vitro* cultures and to evaluate their suitability for preclinical therapeutic assays.

## Results

### Establishment of tumour cell monolayers and 3-D tumour spheres from surgical material

We obtained fresh primary brain tumour tissues from 18 paediatric patients, including 10 medulloblastomas (MB), 1 atypical rhabdoid tumour (ATRT), 2 anaplastic astrocytomas (AA), 2 anaplastic ependymomas (AEP), 1 ependymoma (EP) and 2 pilocytic astrocytomas (PA), and attempted to establish monolayer cultures and 3-D sphere cultures in parallel from these samples. For a summary of patient-related information, see Table 1. In brief, single-cell dissociated cells were seeded in serum-free cell culture medium supplemented with growth factors (referred to as UC, see Material and Methods) in cell culture flasks or 6-well plates without adherence (Ultra-Low™) to enable spheroid formation, or in culture flasks or 6-well plates with adherence (Cell-Bind™) to force cells to grow as adherent monolayers.

Due to the varying volumes of tissues obtained from surgery, the yields of tumour cells from surgical material were highly variable and not comparable between individual tumours. However, even from small tissue samples, >5 × 10^6^ viable cells could generally be obtained from each tumour (see tumour cell counts in Table 1). The loose tissue texture and high tumour cell density of medulloblastomas made it possible to obtain a large number of viable cells from small tissue samples, while other tumour types contained more debris and stromal cells, which made it difficult to estimate the true number of pure tumour cells. However, approximately 3 × 10^6^ cells were seeded per T75 cm^2^ flask or 400 000 cells per 6-well. Fresh medium was added every 3–4 day and cells were passaged when reaching high confluence (adherent cells), or when dark spots (a sign of cell-death due to lack of nutrition) appeared in the spheroids. When cells were transferred to new cell culture vials, at least 1/3 of conditioned medium was added.

### All tumour types have sphere-forming capacity on low-adherence surface, but only subsets of high-grade tumours can be propagated as spheres

**Figure 1:**
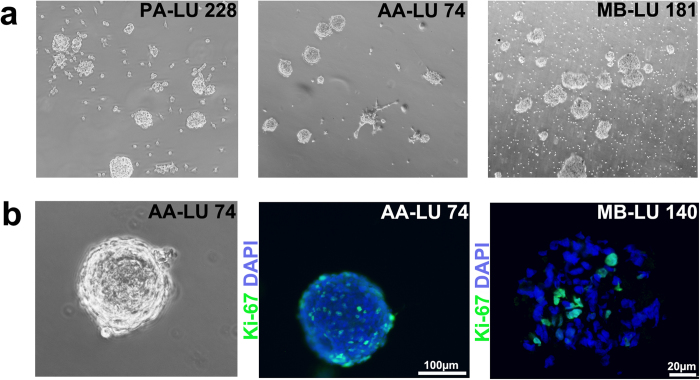
Sphere-forming capacity and propagation of paediatric brain tumour spheres on a low-adherent surface (Ultra-Low™). (**a**) Single-cell dissociated tumour cells, despite grade of malignancy, formed spheres in Ultra-Low™ 6-well plates when seeded at a concentration of 200 000 cells/ml of UC. Representative pictures of a pilocytic astrocytoma (PA-LU 228, WHO grade I), an anaplastic astrocytoma (AA-LU 74, WHO grade III) and a medulloblastoma (MB-LU 181, WHO grade IV) are shown. (**b**) Representative pictures of tumour cell proliferation, as determined by labelling of Ki-67 (green), within a tumour sphere. Anaplastic astrocytoma (AA-LU 74); light microscopy picture, labelling of Ki-67 on tumour spheres attached to a multi-chamber cell culture slide, scale bar is 100 μm. Medulloblastoma (MB-LU 140); labelling of Ki-67 on sectioned tumour spheres, scale bar is 20 μm. DAPI (blue) was used to stain the nuclei.

Traditionally, the neurosphere assay has been utilized as a culture method to preserve the TIC population of the original tumour. When single-cell dissociated tumour tissue was seeded in UC on Ultra-Low™ adherent surface, all tumours (18/18) initially formed spheres after 1–5 days in culture, despite grade of malignancy (Fig. 1a), if seeded at high confluence (200 000 cells/ml). However, only 65% (11/17) of tumours could be further propagated (>passage 3) as sphere cultures (Table 1). Primary spheres obtained from grade I-II tumours did not proliferate, had a loose consistency and eventually dissociated. Upon passaging, no secondary spheres were formed. In contrast, spheres derived from subsets of medulloblastomas, anaplastic astrocytomas and ependymomas were highly proliferative (Fig. 1b) and could be passaged for >3 generations (see Table 1), cryopreserved and thawed for further propagation and analyses. Over multiple passages, spheres were however difficult to dissociate and consequently difficult to count and seed for assays.

### High confluence seeding in UC medium on adherent surface enables cell culture establishment from most primary brain tumour subsets

**Figure 2:**
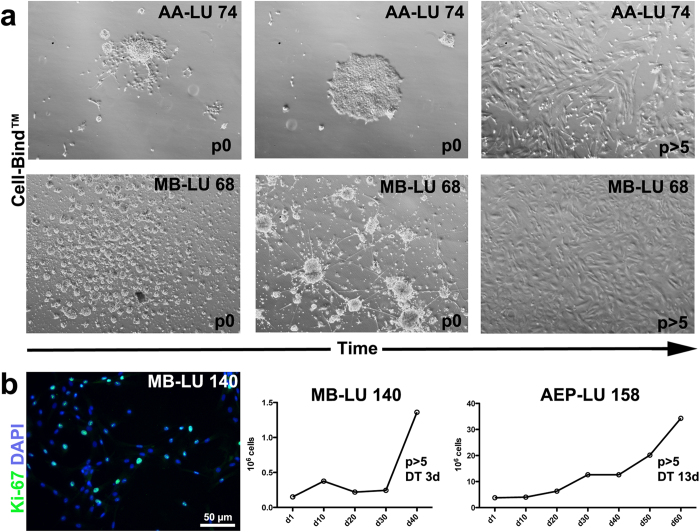
Establishment of proliferating paediatric brain tumour cells on an adherent surface (Cell-Bind™). (**a**) Single-cell dissociated tumour cells and tumour spheroids (either transferred from Ultra-Low 6-well plates, or spheres formed in Cell-Bind™ plates) were forced to attach by culturing in UC on an adherent surface (Cell-Bind™). Cells were passaged when attached spheres flattened on the surface, as illustrated by the middle pictures. Monolayer cultures were formed after passaging the cells multiple times. Representative pictures of an anaplastic astrocytoma (AA-LU 74) and a medulloblastoma (MB-LU 68). (**b**) Representative picture of proliferating adherent medulloblastoma cells (MB-LU 140), as determined by Ki-67 labeling (green). Scale bar is 50 μm. DAPI (blue) was used to stain the nuclei. Proliferation of adherent paediatric brain tumour cells (d1 >passage 5) were determined by counting the number of cells between passages. Cells were either seeded in T-75 Cell-Bind™ flasks (anaplastic ependymoma, AEP-LU 158) or in Cell-Bind™ 6-well plates (medulloblastoma, MB-LU 140). Doubling-time (DT) was calculated at growth-phase (between day 50–60 for AEP-LU 158, between day 30–40 for MB-LU 140).

By seeding primary tumour cells on Cell-Bind™ surface, monolayer cultures were successfully established (>passage 5) from 14/18 (78%) primary tumours, including medulloblastomas, grade II-III ependymomas, high-grade atrocytomas and atypical rhabdoid tumour, see Table 1 and Fig. 2a. High-grade tumours initially formed spheres even in Cell-Bind™ plates, but spheres attached after some time in culture. For medulloblastomas, it took up to several weeks for spheres to attach and generate monolayer cultures. 2 medulloblastomas (molecularly characterized as group 3 and group 4 respectively^32^) did not attach, and could only be propagated as sphere cultures. 4 tumours (betacatenin-activated medulloblastoma, anaplastic astrocytomas/ependymomas) could be propagated as monolayers, but not as spheres. 2/18 tumours, both diagnosed as grade I pilocytic astrocytomas, did initially attach to the plate surface, but did not proliferate for >3 passages. These cell cultures could only be further propagated following addition of FBS to the cell culture medium (data not shown).

Proliferation of adherent brain tumour cells (>passage 5) was demonstrated by Ki67 labelling, and assessed by cell counting between passages. Although confluent cultures contained a substantial amount of proliferating cells (Fig. 2b), tumour cells did not generate colonies when seeded at low densities (1–100 cells/96 well plates). Proliferation rate varied between tumour samples, but was generally slow and fluctuated over time. Doubling time in exponential growth phase was estimated to 3 and 13 days respectively for a fast-growing and a slow-growing cell culture respectively (MB-LU 140, AEP-LU 158, see Fig. 2b).

Although cells were generally cultured for 1–2 months before being cryopreserved, several monolayer cell lines (AA-LU 74, MB-LU 140, AEP-LU 149, AEP-LU 158, MB-LU 159) have been passaged for >20 generations at our lab, and frozen and thawed at multiple occasions without apparent effects on cell viability. Ideally, cell cultures should be established from primary material and passaged for 2–3 passages before being cryopreserved. If cells are single-cell dissociated at surgery and frozen without preculturing, they are more difficult to thaw and propagate at a later occasion.

### Sphere cultures and adherent tumour cell cultures maintain the phenotypic characteristics of the original tumours

**Figure 3:**
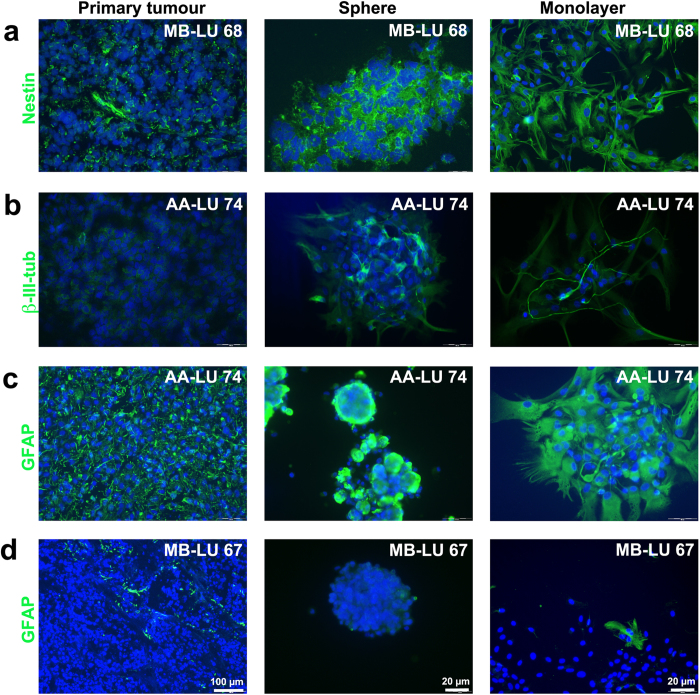
Immunofluorescent staining of neural markers in primary tumours, sphere cultures and monolayer tumour cell cultures. Representative pictures of tumour sections, spheres and monolayer cells immunolabelled (green) with antibodies against (**a**) nestin, (**b**) β-III-tubulin and (**c**, **d**) GFAP. Scale bars are 20–100 μm. DAPI (blue) was used to stain the nuclei.

**Figure 4:**
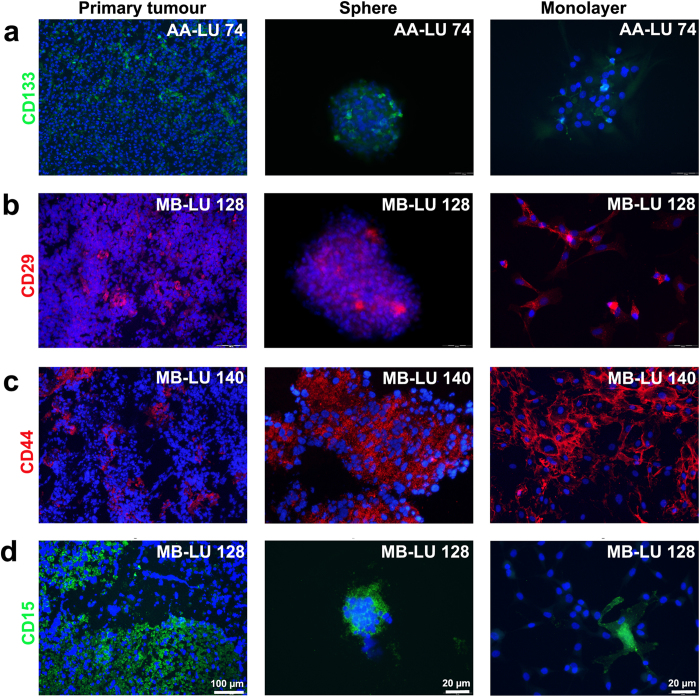
Immunofluorescent staining of CD markers in primary tumours, sphere cultures and monolayer tumour cell cultures. Representative pictures of tumour sections, spheres and monolayer cells immunolabelled with antibodies against (**a**) CD133 (green), (**b**) CD29 (red), (**c**) CD44 (red) and (**d**) CD15 (green). Scale bars are 20–100 μm. DAPI (blue) was used to stain the nuclei.

Serum-cultured adherent glioma cell lines phenotypically diverge from their matched sphere counterparts^6^. To determine if monolayer paediatric brain tumour cells maintained their original phenotype *in vitro*, we used immunofluorecent labelling to investigate the expression of a panel of neural (nestin, nf-200, β-III-tubulin and GFAP) and putative tumour progenitor/stem cell markers (CD15, CD29, CD44 and CD133) in subsets of tumour monolayer cultures. In comparison we labelled the corresponding primary tumours (cryosections) and tumour-derived sphere cultures, and qualitatively scored expression of markers as homogenously or heterogeneously positive (see Table 2). Except for CD15, immature and mature markers were generally not enriched by either culturing method, but were rather expressed in a tumour specific fashion in both spheres and adherent cultures (Table 2 and representative images in Figs 3 and 4).

The neural stem cell marker nestin was highly expressed in all *in vitro* cultures (Fig. 3a), suggesting an immature phenotype of cultured cells. Neuronal maturation markers (nf-200 and β-III-tubulin) were however detected in subpopulations of cells within spheroids, as well as on occasional cells in adherent cultures (Fig. 3b), implying that some degree of *in vitro*-induced cell differentiation will occur regardless of culturing method. Primary tumours expressing the astrocyte marker GFAP maintained the expression in corresponding monolayer and sphere cultures (Fig. 3c), whereas GFAP negative primary tumours remained negative in cell cultures (Fig. 3d). CD29 and CD133 were expressed by subpopulations of cells, both *in vivo* and *in vitro* (Fig. 4a,b). In contrast, CD44 was expressed by a majority of astrocytoma cells (*in vivo*) and ependymoma cells (*in vitro* and *in vivo*). In the medulloblastoma cryosection, CD44 was less prominent, but both sphere and adherent cell cultures were enriched for CD44 (Fig. 4c). Finally, 2/18 tumours in our cohort (MB-LU 159 and MB-LU 72) displayed nuclear β-catenin staining in cryosections. Both tumours maintained nuclear β-catenin on adherent cells (data not shown).

In contrast to all other markers investigated, CD15 was strictly associated with sphere cultures and no or only occasional cells expressed CD15 in adherent cell cultures (Fig. 4d). 5/7 sphere cultures were highly enriched for CD15, even when CD15 was not expressed in the primary tumour (MB-LU 159). Spheres derived from MB-LU 70 and AA-LU 74 were CD15 negative, as were the primary tumours. The pilocytic astrocytomas (PA-LU 226 and PA-LU 228), which could not be propagated *in vitro* in serum-free medium, displayed high expression of CD15 in cryosections (Supplementary Fig. S1).

### Monolayer tumour cells cultured in UC medium express PGE_2_ and its rate-limiting enzyme COX-2 and are suitable for functional assays

**Figure 5:**
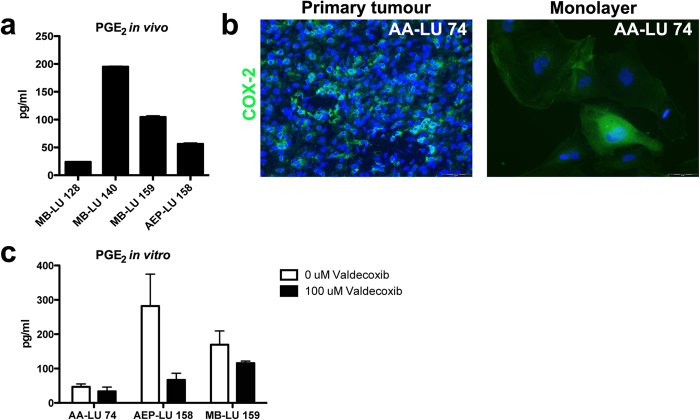
Expression of PGE_2_ and its rate-limiting enzyme COX-2 *in vivo* and by monolayer tumour cells *in vitro*. (**a**) PGE_2_ levels in plasma from patients with medulloblastoma (MB-LU 128, MB-LU 140, MB-LU 159) and anaplastic ependymoma (AEP-LU 158) was analyzed with ELISA. Bars and error bars represent mean and SEM. (**b**) Tumor sections and monolayer cells immunolabelled (green) with antibodies against cycloogygenase-2 (COX-2), the rate-limiting enzyme for prostaglandin E-2 (PGE_2_) production. (**c**) 100 000 monolayer tumour cells were cultured for 24 hours with and without the presence of the COX-2 inhibitor Valdecoxib (100 μM), and supernatants were analysed with ELISA. Bars and error bars represent median and range, measured in three separate experiments for each cell line.

COX-2 is the rate-limiting enzyme for production of the key immunosuppressive factor prostaglandin E_2_ (PGE_2_), and we have previously developed immunotherapeutic treatment strategies including COX-2 inhibition in experimental brain tumour models^27^,^28^. PGE_2_ is detected in plasma isolated from paediatric brain tumour patients at the time of surgery (MB-LU 128, MB-LU 140, MB-LU 159, AEP-LU 158; Fig. 5a), suggesting that paediatric patients could benefit from such a treatment approach.

To investigate the functional utility of our newly established monolayer cultures for preclinical assays, we first demonstrated COX-2 expression by immunolabelling of primary tumours and corresponding *in vitro* cultures from medulloblastomas, anaplastic astrocytoma and anaplastic ependymoma. As shown in Fig. 5b and Table 2, COX-2 expression was detected in all tumour specimens and in the corresponding cell cultures. Next, supernatants from monolayer cell cultures established from three paediatric patients was analysed with ELISA. PGE_2_ was secreted at substantial levels *in vitro* by one ependymoma and one medulloblastoma culture, and secretion was considerably reduced upon treatment with the selective COX-2 inhibitor Valdecoxib (Fig. 5c; AA-LU 74, *p *= 0.25; AEP-LU 158, *p *= 0.13; MB-LU 159, *p* = 0.25 by Wilcoxon matched pairs test). Notably, the experiment was performed at three independent timepoints where cells were cryopreserved and thawed in between, with only small variance in PGE_2_ levels between experiments.

### Cytokine profiling of tumour tissues and monolayer tumour cells

To further assess the inflammatory fidelity of monolayer cells to primary tumours, we characterized the secretory profile of cultured medulloblastomas (*n *= 3), astrocytoma (*n *= 1) and ependymomas (*n *= 2), using a high-sensitivity cytokine multiplex assay (Meso Scale Discovery). 100,000 monolayer cells were cultured in 1 ml UC medium between 24 hours and 7 days, and supernatants were collected and analysed for the presence of IL-6, IL-8, IL-15, IL-16, TNF-α and VEGF, which are known mediators of angiogenesis, proliferation, inflammation and immune suppression. In parallel, we characterized the secretome of lysed primary tumour tissue (encompassing both stromal and tumour cells) obtained from two tumour patients (AEP-LU 158 and MB-LU 140). Measurements were performed three times between passage 5 and 15 for each tumour culture.

**Figure 6:**
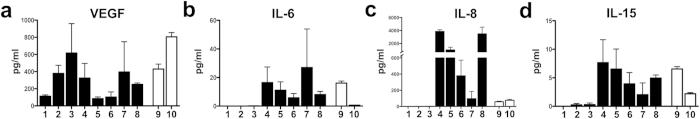
Cytokine profiling of primary tumour tissue and monolayer tumour cells *in vitro*. (**a**–**d**) Cytokine levels in monolayer cell supernatants and tumour tissue lysates were measured with multiplex cytokine assays. Bars and error bars represent mean and SEM of three separate experiments. 1, AA-LU 74 (24 h); 2, AA-LU 74 (48 h); 3, AA-LU 74 (72 h); 4, AEP-LU 149 (48 h); 5, AEP-LU 158 (48 h); 6, MB-LU 69 (48 h); 7, MB-LU 70 (48 h); 8, MB-LU 140 (48 h); 9, AEP-LU 158 (tissue); 10, MB-LU 140 (tissue).

All *in vitro* cell cultures and tumour tissues highly expressed vascular endothelial growth factor (VEGF) (Fig. 6a). The medulloblastoma and ependymoma cultures and tissues also secreted IL-6, IL-8 and IL-15, while the astrocytoma culture secreted no or very low levels of these interleukins (Fig. 6b–d). IL-16 and TNF-α were undetectable in all cell culture supernatants (data not shown), but was secreted in both tumour tissues (IL-16: 5337 ± 33 and 3121 ± 94 pg/ml respectively, TNF-α: 0.30 ± 0.11 and 1.07 ± 0.17 pg/ml respectively). Although the exact quantities of cytokines secreted by tissues and cells were not comparable in this experimental setting, it should be noted that the levels of IL-8 was up to 50x higher in cultured cell samples than in a single tissue samples while levels of other cytokines remained comparable (Fig. 6c). IL-6 secretion in MB-LU 140 tissue could be detected, but was below the lower quantitative limit of this assay (Fig. 6b).

## Discussion

There is currently a lack of clinically relevant *in vitro* models of paediatric brain tumours, as well as comprehensive culturing protocols to generate such models. A recent review of the literature estimated the number of established paediatric brain tumour cell lines to approximately 60, of which only a handful are used in a majority of publications^33^. Here, we describe a standardized protocol for establishing and propagating primary paediatric brain tumour cell cultures, by utilizing a simple serum-free cell culture medium and cell culture plastics with high adherence. This approach enabled cell culture establishment from most primary brain tumour subsets, including ones that failed to generate sphere cultures. Monolayer cells could be robustly passaged for an extended time period, displayed phenotypic similarities with the original tumours, and expressed clinically relevant inflammatory factors *in vitro*.

The traditional approach to establish primary brain tumour cultures as neurospheres is not always successful. Certain brain tumour types, including low-grade tumours and distinct subgroups of high-grade tumours^11^,^12^ have proved to be particularly unsuitable for sphere growth. It has been suggested that sphere-forming ability reflects high tumour stem cell content and poor prognosis in brain tumour patients^12^,^34^. Similarly, we observed that although all tumours initially formed spheres in high-density cell cultures, ‘spheres’ derived from low-grade astrocytomas, ependymomas and a beta-catenin activated medulloblastoma (associated with good prognosis^35^) could not be serially passaged, and initial sphere formation likely reflected fusion rather than sphere-forming ability. In contrast, monolayer cell cultures were successfully established from 78% of primary paediatric brain tumours, including the ones that would not generate neurosphere cultures. This culturing approach could thus be especially useful in establishment of cell cultures from less aggressive tumours, which may otherwise be difficult to propagate in serum-free medium. While monolayer tumour cells grew slowly in serum-free medium compared to traditional serum-cultured cell lines, they could be expanded into numbers sufficient for *in vitro* assays, xenografting in immunodeficient mice or therapeutic immunizations, in approximately 2–3 months. Notably, most medulloblastomas in our cohort also generated robust sphere cultures, and two medulloblastomas (group 3 and 4 respectively^32^) in our cohort could only be propagated as spheres and did not attach to poly-L-lysine-coated plastic (data not shown) or Cell-Bind™. The proneness of primary glioblastomas to generate monolayer and sphere cultures respectively has been reported to reflect molecular subgroup^11^ and the same may be true for medulloblastomas.

While the neurosphere assay has long been considered the golden standard for preserving the TIC population of the primary tumour, recent data has shown that adherent brain tumour cultures maintain tumorigenic capacity when cultured in serum-free medium^16^,^36^. Attempts have been made to culture adherent stem and tumour cells on different attachment substrates, such as laminin, collagen and poly-lysine. The choice of substrate dictates cell fate, and the optimal substrate for maintaining monolayer tumour stem and progenitor cell cultures is yet to be determined. While coating with laminin maintains NSCs *in vitro*^15^, other substrates induce neuronal differentiation of stem cells^37^. Here, we have utilized cell culture flasks or 6-well plates with high adherence plastic (Cell-Bind™), which is a cheap and standardized alternative to biological coating. Although the current study does not provide conclusive evidence for preservation of TICs, we find that our monolayer cultures display extensive self-renewal capacity and are immunophenotypically similar to sphere cultures over serial passages. Additional studies should be conducted to assess if cells also maintain tumorigenic capacity *in vivo*.

CD15 has previously been suggested to be a marker for TICs in adult glioblastomas, ependymomas and medulloblastomas^38^,^39^,^40^. Interestingly, we find that CD15 is highly enriched on most cultured tumour spheres even when it is not expressed in the primary tumour, suggesting upregulation following sphere formation. In contrast, low malignant pilocytic astrocytoma cryosections displayed large areas of intense CD15 labelling, but corresponding cultures did not form spheres in culture. In consistency with our findings, previous studies have also demonstrated an intense CD15 labelling in low malignant human brain tumours, and a lack of CD15 positivity in subsets of high malignant brain tumours^38^,^40^,^41^. In addition, we found that monolayer cells were generally CD15 negative, but did express nestin and did not display considerable expression of maturation markers compared with corresponding spheres. The most frequently used CD15 antibody clones in the literature (Hi98, MC-480 and MMA) react with the cell surface carbohydrate epitope 3-fucosyl-N-acetyllactosamine, which is expressed not only on NSCs and potential TICs, but also on mature human astrocytes and neurons^42^,^43^. Altogether, these findings question the role of CD15 alone as a robust TIC marker in human brain tumours, and also indicate that alterations in CD15 expression could be related to adherence in addition to cellular maturation state.

Immunotherapeutic approaches for malignant brain tumours have shown limited clinical efficacy, despite promising experimental results, partly due to the poor resemblance between preclinical models and primary tumours. *In vitro* culturing in serum-containing medium has been shown to change the immunological phenotype of brain tumour cells, including upregulation of MHC class I and ICAM-1, and altered cytokine secretion^44^. In the current study, we have utilized Lonzas Ultra-Culture serum-free medium, which has previously been shown to preserve tumour antigens of primary human glioma cells to be utilized for clinical immunotherapy^7^. We screened a subset of monolayer cultures for secretion of a panel of inflammatory factors, and compared to the secretory profile of fresh frozen unsorted tumour tissue comprising both tumour and stromal cells. All brain tumour cultures investigated in this study displayed high levels of VEGF and PGE_2_, which are well known therapeutic targets and mediators of angiogenesis and immune suppression in malignant paediatric brain tumours^45^,^46^. We also report IL-15 secretion by brain tumour cells *in vivo* and *in vitro*. IL-15 is ubiquitously expressed in the brain and promotes neuron survival and neuroinflammation in multiple sclerosis and experimental autoimmune encephalomyelitis^47^,^48^, although its functions in CNS malignancies are not known. Moreover, we detected substantial secretion of IL-8 in medulloblastoma and ependymoma tissues and cultures, confirming previous reports of IL-8 expression in these tumour types *in vivo*^49^. The quantitatively high overall levels of IL-8 *in vitro* however indicate that IL-8 secretion may be partly enhanced by cell culturing. The same could be true for IL-6, which was detected in one medulloblastoma cell culture where IL-6 was not detected at significant levels in the corresponding primary tumour. Finally, IL-16 and TNF-α were secreted at high levels in tumour tissues, but were undetectable in all tumour cultures, in consistency with reports that IL-16 and TNF-α expression in human and mouse gliomas predominantly originates in tumour-associated macrophages/microglia^50^,^51^.

Regardless of culture method, tumour cells indisputably change when transferred to *in vitro* conditions. Concerns have been raised whether adherent brain tumour cell lines in serum-free conditions are equivalent to corresponding sphere cultures^52^, but experimental evidence is scarce at this point. Nevertheless, monolayer cell culturing is a standard method at most laboratories and the practical benefits of using adherent cells rather than spheres are easily appreciated. Our results so far show that this is a feasible method for establishment, propagation and assessment of paediatric brain tumour cells, and encourages further molecular studies of serum-free monolayer cultures.

## Methods

### Patients and tumour sampling

All experiments were performed in accordance with national regulations and were approved by the Local Ethical Review Board of Lund, Sweden (ETIK2008/642) and the Ethical Research Board of the Medical Faculty at Lund University, Lund, Sweden (serial number LU1028-03). Brain tumour and blood samples were obtained from 18 paediatric patients (<18 years) at the time of surgical resection, and tumours were histologically classified according to WHO criteria. For a summary of patient information, see Table 1. All patients and/or their parents gave their informed consent prior to inclusion in the study.

### Tumour dissociation and cell culturing

Parts of the fresh tumour tissue was prepared for mesoscale analysis or cryosectioning, see below. The remaining part of the tumour material was single-cell dissociated for cryopreservation and cell culturing. Tumours were cut in small pieces using a sterile scalpel-blade in a petridish containing Accutase® (Sigma-Aldrich, Stockholm, Sweden) or TrypLE™ Express (Gibco®, Life Technologies, Stockholm, Sweden) and incubated for 15 minutes at 37 °C. Tumours were then further dissociated in 5 ml cell culture medium by pipetting using a 5 ml serological pipet, and mechanically minced through a BD Falcon™ cell strainer (75 μm) (BD Biosciences Pharmingen, Stockholm, Sweden) using a 2 ml syringe plunger. Cell culture medium was used to rinse the strainer to approximately 8 ml in total of cell suspension. Cells were centrifuged for 8 min at 1200 rpm. The supernatant was removed and cells were resuspended in medium and counted.

Approximately 3 × 10^6^ cells were transferred to a T75 75 cm^2^ cell culture flask, Cell-Bind™ — adherent plastic (Corning, VWR, Stockholm, Sweden) or Ultra-Low™ — non-adherent plastic (Corning, VWR). In parallel, approximately 400 000 cells/well were transferred to a 6-well plate (Cell-Bind™, Ultra-Low, Corning, VWR). Cells were incubated at 37 °C, 21% O_2_ and 6% CO_2_ and fed with fresh cell culture medium; UltraCULTURE™ cell culturing medium (Lonza BioWhittaker Inc., VWR) supplemented with 2 mM L-glutamine (Lonza BioWhittaker Inc., VWR), 1% antibiotics (Penicillin-Streptomycin, Life Technologies), bFGF (40 ng/mL medium, Chemicon, Merck Millipore cat # GF003, Solna, Sweden) and EGF (20 ng/mL medium, Chemicon, Merck Millipore cat # GF144), every 3–4 day. Cell culture medium will be referred to as UC. Tumor cells cultured non-adherently formed tumour spheres and were either passaged mechanically by trituration using a 1000-200 μl pipett or enzymatically using Accutase® (Sigma-Aldrich) or TrypLE™ Express (GIBCO®, Life Technologies). Adherent cells were passaged by rinsing in PBS without Ca2+ and Mg2+ (GIBCO® Life Technologies) and incubation with Accutase® (Sigma-Aldrich) or TrypLE™ Express (GIBCO®, Life Technologies) at 37 °C for 5–15 minutes. Passaged cells were resuspended in 2/3 fresh UC medium and 1/3 conditioned medium.

### Cryopreservation of tumour cells

Single-cell dissociated fresh tumour tissue and cultured tumour cells at different passages were pelleted, resuspended and frozen in freezing medium; 1,5 ml UC medium w/o growth-factors, and antibiotics, supplemented with 70% foetal bovine serum (FBS) (Biochrom,AB, Berlin, Germany), 10% dimethyl sulfoxide (DMSO) (Sigma-Aldrich). Cell vials were placed in a Mr.Frosty™ (ThermoScientific, Gothenburg, Sweden) containing isopropanol (Sigma-Aldrich) at –80 °C for 24 hours before long-term storage in liquid nitrogen for later use.

For thawing, cell vials were held in running warm water until the frozen pellet disappeared. The cell suspension was transferred to 2 ml cold UC and kept on ice for 5 min. 2 ml cold UC was added to the cell suspension, and cells were left on ice for another 2 min., followed by a final addition of 5 ml cold UC. Cells were centrifuged for 8 min. at 1200 rpm, resuspended in UC and seeded in cell culture vials as described above.

### Proliferation and clonogenicity assays

Adherent tumour cells >p5 were seeded in either T75 75 cm^2^ Cell-Bind™ cell culture flasks at a concentration of 3–4 × 10^6^ cells/10 ml UC, or in 6-well Cell-Bind™ cell culture wells at a concentration of 150 000-400 000 cells/2 ml UC. Cells were passaged as described above, counted every 10 days for 40 to 60 days and seeded in new cell culture vials. Doubling-time was calculated according to the following formula; DT=Tln2/ln(number of cells end/number of cells start) where DT is doubling-time, and T is total number of days.

To test clonogenic capacity of cultured cells, single-cell dissociated adherent tumour cells (AA-LU 74, AEP-LU 158 and MB-LU 159) at passage >15 were diluted to 100, 10 and 1 cell/200 μl and plated in Cell-Bind™ 96-well plate (>24 wells/dilution) (Corning, VWR). Numbers of colonies were recorded after >3 weeks.

### Immunofluorescent labelling of tumour cryosections and cultured cells

Fresh tumour tissue was snap-frozen in isopentane, sectioned and stained as previously described^53^. For immunofluorescent labelling of cultured tumour cells, 8-well multichamber slides (BD Biosciences) were coated with poly-L lysine (GIBCO®, Life Technologies) for 4 h, and spheres (>p3) or adherent cells (>p5) attached overnight in a cell culture incubator. Cells were fixed in 4% paraformaldehyde for 30 min, incubated with 0.3% Triton-X100 for 5 min when staining for intracellular markers, blocked with 5% goat serum (DAKO, Stockholm, Sweden) and incubated with primary antibodies for 2,5 h at 37 °C. Finally, cells were labelled with a secondary antibody for 30 min and mounted with ProLong Gold anti fade containing DAPI (Molecular Probes, Life Technologies). Alternatively, tumour spheres were pelleted, embedded in Cryomount (Histolab Products AB, Gothenburg, Sweden), sectioned and stained as described for cryosections.

Primary antibodies: mouse anti GFAP (5 μg/ml), mouse anti human CD15, clone HI98 (diluted 1:10), PE-mouse anti human CD29 (diluted 1:10), PE-mouse anti human CD44 (diluted 1:10) (BD Biosciences), rabbit anti nestin, (5 μg/ml) rabbit anti COX-2 (5 μg/ml), rabbit anti β-catenin (4 μg/ml) (Abcam, Cambridge Science Park, Cambridge, UK), rabbit anti Ki67 (diluted 1:40, Biotrend, Köln, Germany), mouse anti CD133/2 (5 μg/ml, Milteney Biotec GmbH, Bergisch Gladbach, Germany), rabbit anti neurofilament 200 (5 μg/ml), mouse anti β-III-tubulin (diluted 1:500) (Sigma Aldrich). For fluorescent labelling, Alexa Fluor 488-goat anti mouse and Alexa Fluor 488-goat anti rabbit (5 μg/ml, Molecular Probes, Life Technologies) were used as secondary antibodies. Matched isotype antibodies were used as negative controls.

### Image analysis

Cryosections and cells were examined using a light microscope (BX-53, Olympus LRI Instruments AB, Lund, Sweden) equipped with a mercury lamp and filters for fluorescence (U-MWG, U-MWB, U-MWU). Images were taken in 10x, 20x or 40x magnification using an Olympus Color View digital camera and captured using cellSens Dimension imaging software (Olympus).

### PGE_2_ detection and Valdecoxib treatment

Blood samples from brain tumour patients (MB-LU 128, MB-LU 140, MB-LU 159, AEP-LU 158) were treated with 10 uM indomethacin (Sigma-Aldrich) and 250 IE/KY/ml heparin (Heparin LEO, 5000 IE/KY/ml, LEO Pharma AB, Malmö, Sweden). Plasma was isolated by centrifugation at 1000 g for 10 min and stored at −80 °C until analysis. PGE_2_ concentrations were measured in duplicates using PGE_2_ EIA kit (Cayman Chemicals, Larodan Fine Chemicals AB, Malmö, Sweden).

100 000 adherent tumour cells (MB-LU 159, AEP-LU 158 and AA-LU 74, p5-p15) were seeded in 1 ml UC medium with or without Valdecoxib (100 μM) for 24 hours in three separate experiments. PGE_2_ concentrations were measured in duplicates using PGE_2_ EIA kit (Cayman Chemicals, Larodan Fine Chemicals AB, Malmö, Sweden), and compared using Wilcoxon matched pairs test (GraphPad Prism 5.0, San Diego, USA).

### Cytokine secretion in primary tumour tissues and cultured cells

Fresh tumour tissue was snap-frozen and stored at −80 °C. Frozen tissue was mechanically homogenized in MSD Tris Lysis Buffer (Meso Scale Discovery, Rockville, MD, USA) and sonicated for 2 min. Following 20 min incubation on ice, samples were centrifuged for 10 min at 2000 g and supernatants were collected and stored at −80 °C until subsequent analysis. 100,000 monolayer tumour cells (AA-LU 74, AEP-LU 149, AEP-LU 158, MB-LU 69, MB-LU 70, MB-LU 140, MB-LU 159, p5-p15) were cultured in 1 ml medium for 24 hours-7 days in three separate experiments and supernatants were collected and stored at −80 °C. Tissue lysates and cell supernatants were analysed in duplicates with high-sensitivity cytokine multiplex assays (Meso Scale Discovery) Cytokine Panel 1 and Proinflammatory Panel 1 according to the manufacturer´s instructions.

## Additional Information

**How to cite this article**: Sandén, E. *et al.* A standardized and reproducible protocol for serum-free monolayer culturing of primary paediatric brain tumours to be utilized for therapeutic assays. *Sci. Rep.* **5**, 12218; doi: 10.1038/srep12218 (2015).

## Supplementary Material

Supplementary Figure S1

## Figures and Tables

**Table 1:** Summary of patient related information and cell culture establishment.

Patient ID	PAD	Grade	Age (yrs)	Gender	DOD	Cell count	3-D spheroids	Monolayer
**MB-LU 67**	MB	IV	12	M	–	>20 × 10^6^	X	–
**MB-LU 68**	MB	IV	10	M	–	>20 × 10^6^	X	X
**MB-LU 69**	MB	IV	9	M	–	>100 × 10^6^	X	X
**MB-LU 70**	MB	IV	8	M	X	>80 × 10^6^	X	X
MB-LU 72*	MB	IV	2	M	X	nd	X	X
**MB-LU 128**	MB	IV	13	M	–	>60 × 10^6^	X	X
**MB-LU 140**	MB	IV	6	M	–	>60 × 10^6^	X	X
**MB-LU 159**	MB	IV	11	F	–	<5 × 10^6^	–	X
**MB-LU 181**	MB	IV	4	M	X	>30 × 10^6^	X	–
**MB-LU 187**	MB	IV	7	M	–	>30 × 10^6^	X	X
**ATRT-LU 315**	ATRT	IV	5	F	–	<5 × 10^6^	nd	X
AEP-LU 149*	AEP	III	14	M	X	>50 × 10^6^	–	X
AEP-LU 158*	AEP	III	14	M	X	>30 × 10^6^	X	X
**EP-LU 78**	EP	II	2	M	–	>100 × 10^6^	–	X
**AA-LU 74**	AA	III	0	F	–	<5 × 10^6^	X	X
AA-LU 221*	AA	III	17	M	–	>30 × 10^6^	–	X
**PA-LU 226**	PA	I	11	F	–	<5 × 10^6^	–	–
**PA-LU 228**	PA	I	14	F	–	24 × 10^6^	–	–

PAD, pathologic anatomic diagnosis; DOD, dead of disease (X indicates DOD); MB, medulloblastoma; ATRT, atypical rhabdoid tumor; AEP, anaplastic ependymoma; AA, anaplastic astrocytoma; EP, ependymoma; PA, pilocytic astrocytoma; MN, meningioma; nd, not determined.

^*^relapse. X indicates cultured for >passage 3 (3D spheroids) or >passage 5 (monolayer cells).

**Table 2:** Immunophenotypes of paediatric brain tumour cryosections and cultured cells.

Tumour	CD15			CD29			CD44			CD133			nestin			nf-200/β-III-tub			GFAP			COX-2		
	T	S	M	T	S	M	T	S	M	T	S	M	T	S	M	T	S	M	T	S	M	T	S	M
MB-LU 67	+	nd	nd	+	nd	nd	nd	nd	nd	+	+	nd	+	++	nd	+	+	nd	−	−	−	+	+	nd
MB-LU 68	+	++	−	+	nd	nd	nd	nd	nd	+	−	nd	+	++	++	+	nd	+	−	nd	−	+	nd	nd
MB-LU 70	−	−	nd	+	−	+	+	+	nd	+	+	nd	+	++	++	−	+	+	+	++	−	+	+	+
MB-LU 128	+	++	+	+	+	+	nd	nd	nd	+	−	nd	+	−	nd	+	nd	nd	+	nd	nd	+	+	+
MB-LU 140	+	++	−	+	+	+	+	++	++	+	+	+	+	++	++	+	+	+	+	++	++	+	+	
MB-LU 159	−	++	−	−	−	−	nd	nd	nd	+	nd	nd	+	++	++	+	+	+	+	++	++	+	+	+
AA-LU 74	−	−	−	+	−	−	++	nd	nd	+	+	+	+	++	++	−	+	+	++	++	++	+	++	+
AEP-LU 158	+	++	−	−	nd	−	++	++	++	+	+	+	+	nd	+	−	nd	−	++	nd	++	+	nd	+

Protein detection by immunofluorescent labelling of tumour cryosections (T, left column), tumour spheres (S, middle column) and monolayer cells (M, right column). ++ indicates homogeneously positive; + indicates heterogeneously positive; − indicates negative; nd, not determined. For MB-LU 159, low-passage spheres (p <3 ) were stained.
