# Supplementary material for: A standardized and reproducible protocol for serum-free monolayer culturing of primary paediatric brain tumours to be utilized for therapeutic assays
Source: Sci Rep. 2015 Jul 17;5:12218. doi: 10.1038/srep12218 (PMC4505308; doi:10.1038/srep12218)
Supplement: Supplementary Figure S1 [file srep12218-s1.pdf]

A standardized and reproducible protocol for serum-free monolayer culturing of primary paediatric brain tumours to be utilized for therapeutic assays

Emma Sandén[1]*, Sofia Eberstål[1], Edward Visse[1], Peter Siesjö[1,2], Anna Darabi[1]

[1]Glioma Immunotherapy Group, Division of Neurosurgery, Department of Clinical Sciences Lund, Lund University, Lund, Sweden

[2]Division of Neurosurgery, Department of Clinical Sciences Lund, Skane University Hospital, Lund, Sweden

A

Patient ID	PAD	%
MB-LU 67	MB	1.13
MB-LU 68	MB	1.13
MB-LU 69	MB	1.20
MB-LU 70	MB	0.13
MB-LU 72	MB	1.03
MB-LU 128	MB	11.81
MB-LU 140	MB	3.07
MB-LU 159	MB	0.85
MB-LU 181	MB	0.65
MB-LU 187	MB	13.29
AEP-LU 158	AEP	18.17
AEP-LU 270	AEP	7.76
EP-LU 78	EP	21.15
AA-LU 74	AA	0.84
AA-LU 221	AA	5.76
PA-LU 226	PA	11.34
PA-LU 228	PA	17.78

B

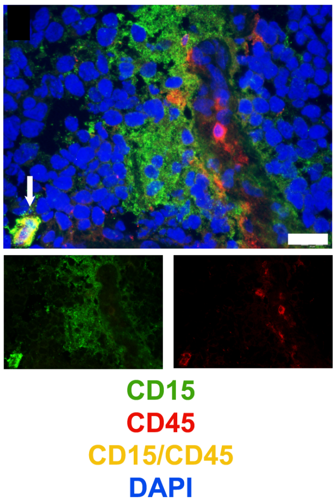

C

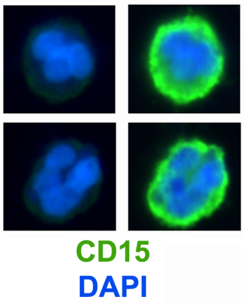

Supplementary Figure S1. CD15 stained area fraction in tumor cryosections. For quantitative assessment of tumor-associated CD15 expression in tissues, cryosections were labeled with mouse anti human CD15, clone HI98 (diluted 1:10, BD Biosciences Pharmingen, Stockholm, Sweden) followed by Alexa Fluor 488-goat anti mouse (5 µg/ml, Molecular Probes, Life Technologies) and PE-mouse anti human CD45 (diluted 1:10, BD Biosciences Pharmingen, Stockholm, Sweden). To obtain images of whole cryosections, a series of images were taken in 20x magnification using an Olympus Color View digital camera (Olympus LRI Instruments AB, Lund, Sweden) and merged with Multi-image alignment. The percentage of CD15+ stained area fraction (**A**) was calculated using cellSens Dimension imaging software (Olympus). CD15 was expressed by neutrophils, identified by CD45 positivity (**B**, arrow) and segmented nuclei (**C**), in addition to tumor cells. CD15+CD45+ cells could however be excluded from quantitative analysis by flourescence tresholds.
